# LipidSigR: a R-based solution for integrated lipidomics data analysis and visualization

**DOI:** 10.1093/bioadv/vbaf047

**Published:** 2025-03-10

**Authors:** Chia-Hsin Liu, Pei-Chun Shen, Meng-Hsin Tsai, Hsiu-Cheng Liu, Wen-Jen Lin, Yo-Liang Lai, Yu-De Wang, Mien-Chie Hung, Wei-Chung Cheng

**Affiliations:** Cancer Biology and Precision Therapeutics Center, China Medical University, Taichung 404328, Taiwan; Cancer Biology and Precision Therapeutics Center, China Medical University, Taichung 404328, Taiwan; Cancer Biology and Precision Therapeutics Center, China Medical University, Taichung 404328, Taiwan; Cancer Biology and Precision Therapeutics Center, China Medical University, Taichung 404328, Taiwan; School of Medicine, China Medical University, Taichung 404328, Taiwan; Department of Radiation Oncology, China Medical University, Taichung 404328, Taiwan; Graduate Institute of Biomedical Sciences, China Medical University, Taichung 404328, Taiwan; Department of Urology, China Medical University, Taichung 404328, Taiwan; Cancer Biology and Precision Therapeutics Center, China Medical University, Taichung 404328, Taiwan; Graduate Institute of Biomedical Sciences, China Medical University, Taichung 404328, Taiwan; Molecular Medicine Center, China Medical University Hospital, China Medical University, Taichung 404328, Taiwan; Department of Biotechnology, Asia University, Taichung 413305, Taiwan; Cancer Biology and Precision Therapeutics Center, China Medical University, Taichung 404328, Taiwan; Graduate Institute of Biomedical Sciences, China Medical University, Taichung 404328, Taiwan; The Ph.D. Program for Cancer Biology and Drug Discovery, China Medical University and Academia Sinica, Taichung 404328, Taiwan

## Abstract

**Motivation:**

Lipidomics is a rapidly expanding field focused on studying lipid species and classes within biological systems. As the field evolves, there is an increasing demand for user-friendly, open-source software tools capable of handling large and complex datasets while keeping pace with technological advancements. LipidSig, a widely used web-based platform, has been instrumental in data analysis and visualization of lipidomics. However, its limitations become evident when users want to build customized workflows. To address the limitation, we developed a companion R package, LipidSigR, based on the R code of the LipidSig web platform.

**Results:**

LipidSigR offers greater flexibility, allowing researchers with basic R programming skills to modify and adapt workflows according to their needs. It has been rigorously tested following CRAN guidelines to ensure compatibility and reproducibility. In demonstrating its functionality, we analyze the case with commonly used experimental design, case versus control, in lipidomics studies. Researchers can follow the use case to explore the key capabilities and build customized lipidomics data analysis workflows using LipidSigR.

**Availability and implementation:**

LipidSigR is freely available from https://lipidsig.bioinfomics.org/lipidsigr/index.html and https://github.com/BioinfOMICS/LipidSigR.

## 1 Introduction

Lipidomics is a rapidly growing field focusing on the comprehensive study of all lipid species and classes within biological systems. This discipline plays a critical role in advancing our understanding of complex biological networks and complements other omics technologies, such as genomics and proteomics, in multi-omics approaches to system characterization (Kopczynski *et al.* 2017, Ahluwalia *et al.* 2022). Consequently, lipidomics is positioned to influence the development of precision medicine significantly (Ni *et al.* 2023). However, with the increasing complexity of lipidomics data, there is an urgent demand for user-friendly, open-source software tools capable of handling large datasets while keeping up with fast-paced technological advancements.

The landscape of lipidomics data analysis is enriched by a wide variety of web tools, including LipidSig (Lin *et al.* 2021, Liu *et al.* 2024), LipidSuite (Mohamed and Hill 2021), LipidOne (Pellegrino *et al.* 2022), and LION/web (Molenaar *et al.* 2019). Among these, LipidSig is one of the most comprehensive tools for statistical and functional analysis in lipidomics. Despite its wide adoption and user-friendly design, the web-based nature of LipidSig poses challenges. Users might often struggle with reanalyzing their data outside the web interface, and the platform’s limitations become apparent when dealing with customized workflows. As lipidomics applications continue to expand across diverse biological systems, it is clear that data analysis cannot rely on a “one size fits all” approach. To overcome the gap, we developed LipidSigR—a companion R package. By releasing the source code for key functions in the LipidSig web server, LipidSigR empowers researchers with basic R programming skills to modify and adapt the tools according to their specific needs in lipidomics data analysis.

## 2 Implementation and features

### 2.1 Implementations

LipidSigR is developed in the R programming language (R Core Team 2024) and is available on GitHub (https://github.com/BioinfOMICS/LipidSigR). It builds upon the foundational R code from the LipidSig web server, with substantial modifications to ensure compatibility and efficiency for command-line use in R. To simplify the learning curve for users, we have created detailed reference documents that provide practical exposure to key R commands (https://lipidsig.bioinfomics.org/lipidsigr/reference/index.html). LipidSigR has undergone rigorous testing to ensure the quality meets the expected standards of R packages (Wickham 2015).

### 2.2 Features

**Figure 1.**
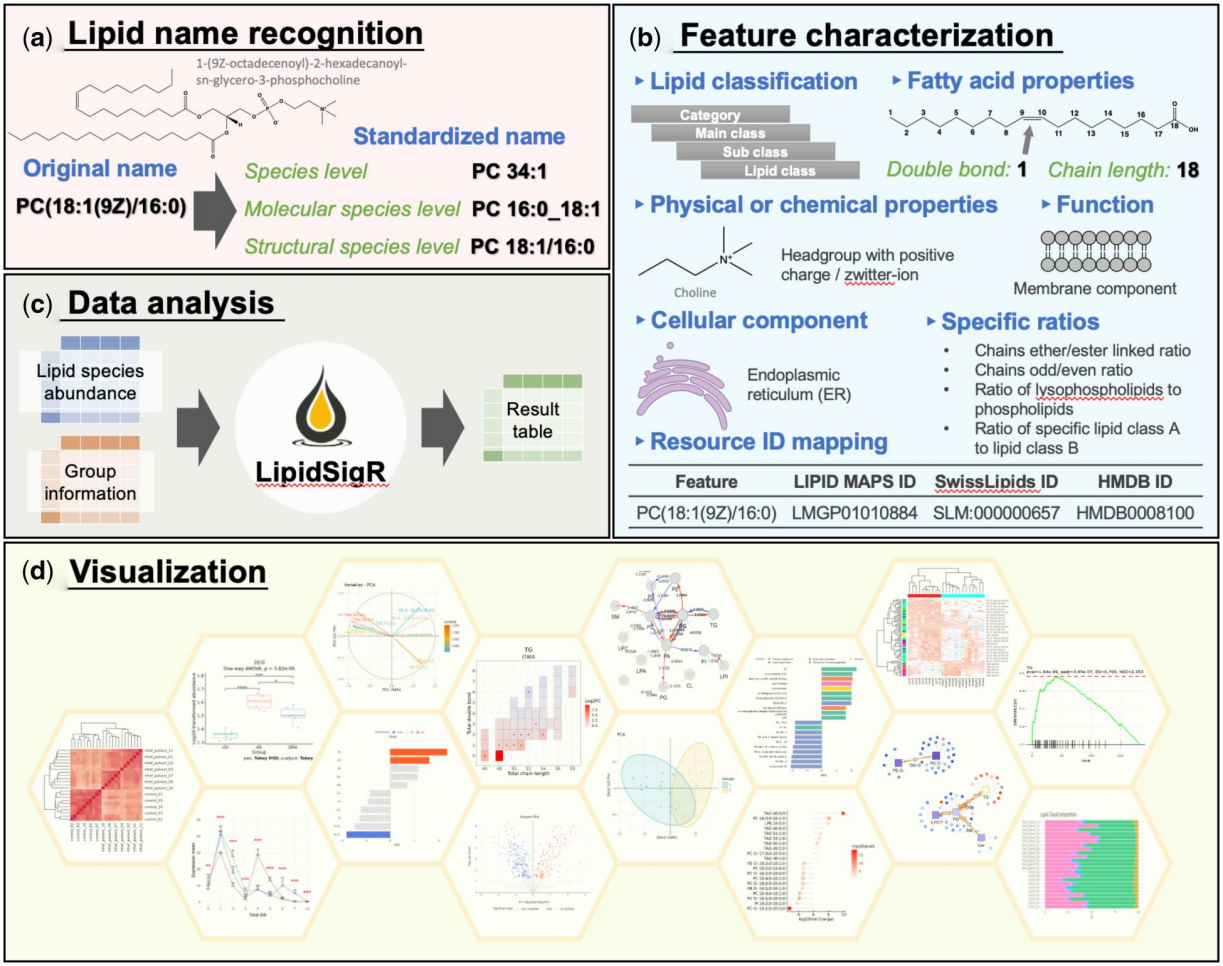
Implement of LipidSigR. This figure illustrates the basic concept of lipidomics data analysis workflow using LipidSigR. From top to bottom, the workflow begins with (a) Lipid Name Recognition, where lipid species are classified and identified based on standardized lipid nomenclature. This is followed by (b) Feature Characterization, which includes the characterization of lipid features such as lipid class, fatty acid chain length, and structural properties. Next, (c) Data Analysis involves the application of statistical and bioinformatic methods to extract meaningful patterns, including differential expression and enrichment analyses. Finally, (d) Visualization presents the analyzed data in various formats, such as heatmaps, principal component analysis plots, and network diagrams, enabling users to gain insights into lipidomic data and their biological significance.

LipidSigR encompasses 48 functions and example datasets, providing capabilities for lipid name recognition, feature characterization, data analysis, and visualization, as depicted in Fig. 1. The software integrates the LipidSig knowledgebase, which includes a robust lipid nomenclature system and an ID conversion framework to standardize lipid name recognition (Fig. 1a). Additionally, LipidSigR incorporates several R packages, such as Goslin (Kopczynski *et al.* 2022) and LION (Molenaar *et al.* 2019), to enable detailed lipid characteristic identification, including fatty acid unsaturation levels, chain length, and other structural properties (Fig. 1b). LipidSigR also provides various data analysis methods (Fig. 1c), including statistical, bioinformatics, and network analysis (see the following use case section for details). Advanced data visualization (Fig. 1d) is supported through ggplot2 (Wickham 2016), enhancing the interpretability of analysis results and facilitating their translation into meaningful biological insights.

The LipidSig web platform has been updated to feature an R command demonstration specifically designed for LipidSigR (https://lipidsig.bioinfomics.org/lipidsigr/index.html). Users can access detailed vignettes and step-by-step tutorials for the demonstration cases. The demonstration codes can be readily copied and pasted into R or RStudio, enabling users to reproduce identical results with minimal effort or modify the workflow for other purposes, even for those with limited programming experience. Notably, the comprehensive documentation, encompassing code and results, is readily available under the designated “Articles” section of the LipidSigR web portal, ensuring the satisfaction of diverse research requirements. Thus, compared to the LipidSig web platform, the LipidSigR package can facilitate users with basic R programming skills to conduct lipidomics analysis or flexibly develop their customized workflow.

## 3 Use case: data analysis and visualization

**Figure 2.**
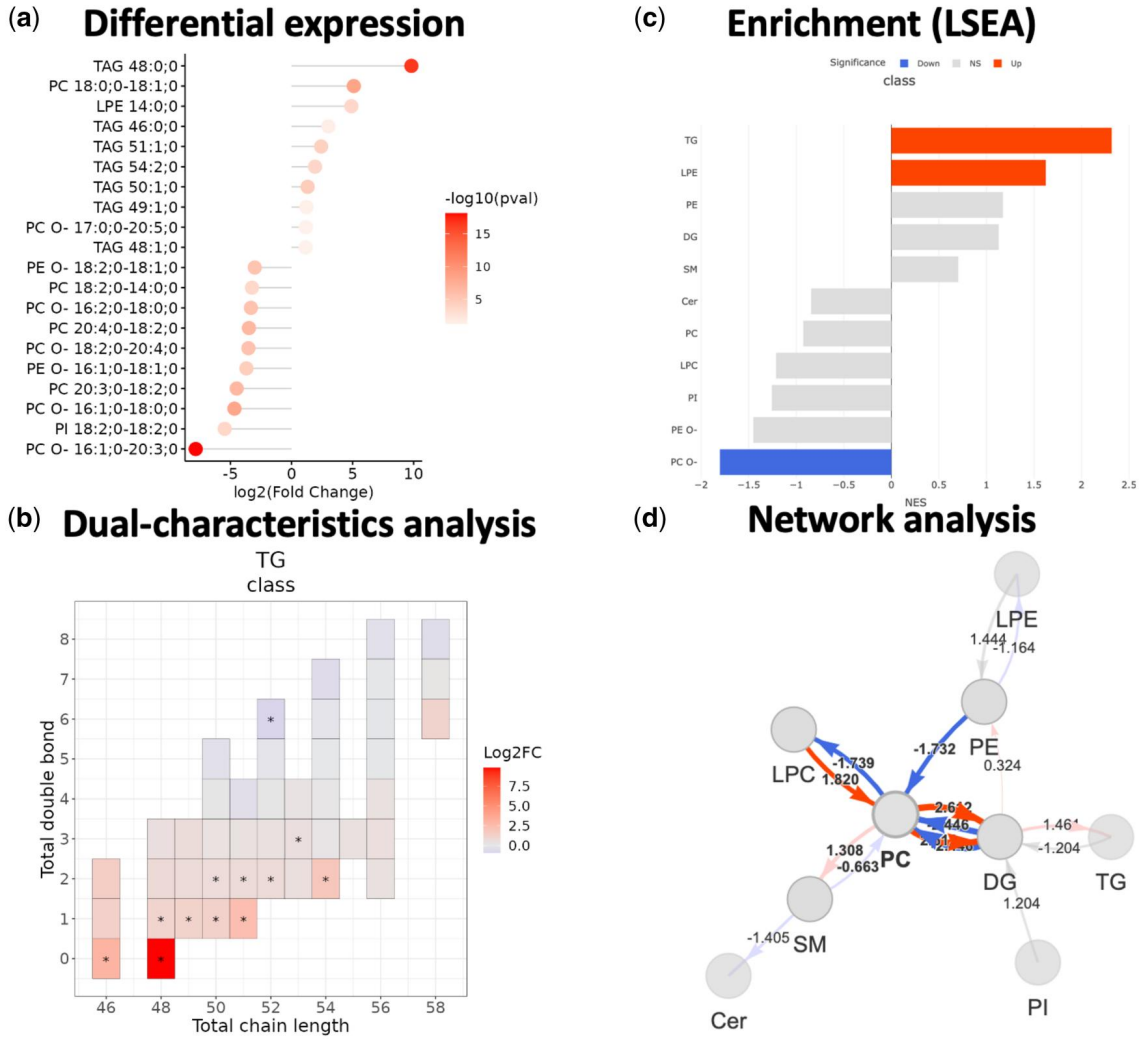
Lipidomic data analysis results and visualizations for the use case. (a) The top 10 differentially expressed lipid species, including both up-regulated and down-regulated lipids, respectively, are depicted in the lollipop plot. (b) The correlation between fatty acid chain length and double bond count within the triacylglycerol (TG) class are depicted in the heatmap. Differentially expressed lipid species with statistical significance between groups are marked with an asterisk (*). (c) Lipid classes enriched with either up- or down-regulated lipids are shown in the bar plot. (d) Activated and repressed lipid activity pathways are depicted in the network diagram. Nodes represent lipid classes, while directed edges indicate pathways, with red signifying activation and blue denoting repression. The thickness of edge represents the score of pathways and absolute scores >1.5 are highlighted in the network diagram.

To illustrate the capabilities and functionalities of LipidSigR, an analysis of the lipidomics data from the study “Adipose tissue ATGL modifies the cardiac lipidome in pressure-overload-induced left ventricular failure” (Salatzki *et al.* 2018) is demonstrated. As analysis results illustrated in Fig. 2, this example provides a clear walkthrough for users to follow, guiding them through the initial analysis steps.

### 3.1 Differential expression

LipidSigR enables users to perform differential expression analyses (https://lipidsig.bioinfomics.org/lipidsigr/articles/3_de.html) to identify lipids that exhibit significant differences between groups, such as HFrEF and healthy controls. For instance, several triacylglycerol (TAG) species, such as TAG 48:0, were found to be upregulated, whilst certain phosphatidylcholine-ether (PC O–) species, such as PC O– 16:1–20:3, were downregulated (Fig. 2a). Furthermore, dual-characteristics analysis has been used to illustrate correlations between fatty acid (FA) chain length and double bond content. This reveals that TAGs with shorter FA chains and lower desaturation levels were significantly regulated in HFrEF (Fig. 2b).

### 3.2 Enrichment analysis

LipidSigR provides support for enrichment analyses, including over-representation analysis (ORA) and lipid set enrichment analysis (LSEA) (Korotkevich *et al.* 2016) (https://lipidsig.bioinfomics.org/lipidsigr/articles/4_enrichment.html) to translate differential expression results into biological insights. These analyses identify biological functions enriched by up- or downregulated lipids, which are then visualized through bar plots. For instance, LSEA demonstrated that up-regulated lipid species in HFrEF were enriched in TAGs, while down-regulated species were enriched in PC O- (Fig. 2c). These findings enhance the understanding of the biological significance and functional context of lipidome remodeling. It is important to note that the enrichment analysis module in LipidSigR is versatile, allowing for the analysis of both the differential expression results generated from the functions in LipidSigR and from user-provided datasets with the requisite columns—such as lipid names, *P*-values, and associated statistics—are included.

### 3.3 Network analysis

LipidSigR enables users to conduct pathway activity network analysis by utilizing differential expression results, generated from the functions in LipidSigR or provided by the user, to assess flux alterations within lipid reaction networks (Gaud *et al.* 2021, Emelianova *et al.* 2022) (https://lipidsig.bioinfomics.org/lipidsigr/articles/5_network.html). This approach identifies pathways that are either actively engaged or suppressed, providing critical insights into the dynamic behavior of lipid metabolism. For instance, pathway activity network analysis revealed decreased reaction activity from phosphatidylethanolamine (PE) to phosphatidylcholine (PC), suggesting a reduced PC/PE ratio in HFrEF (Fig. 2d). This alteration, crucial for maintaining membrane integrity, may increase membrane permeability and contribute to cellular damage. These features greatly enhance the understanding of the biological significance of lipidomic alterations.

### 3.4 Profiling

In addition to the previously demonstrated use case, the “Profiling” module in LipidSigR facilitates comprehensive exploration of lipidomics datasets through a variety of functions (https://lipidsig.bioinfomics.org/lipidsigr/articles/2_profiling.html). For example, users can perform dimensionality reduction analyses, including principal component analysis (PCA), t-distributed stochastic neighbor embedding (t-SNE), and uniform manifold approximation and projection (UMAP), to investigate cross-sample variability. Furthermore, users can also conduct correlation analyses to reveal intrinsic correlation patterns among samples.

## 4 Conclusion

Data analysis remains a critical bottleneck in modern lipidomics workflows. Conducting thorough and detailed analyses of lipidomics data presents challenges for many researchers, necessitating robust and adaptable software solutions. LipidSigR addresses these needs by complementing the widely used LipidSig web server, providing a versatile R package that empowers researchers to perform sophisticated lipidomics data analyses and visualizations.

## Data Availability

LipidSigR is freely available from https://lipidsig.bioinfomics.org/lipidsigr/index.html and https://github.com/BioinfOMICS/LipidSigR.
